# Carbapenemase-producing Enterobacteriaceae colonisation in adult inpatients: A point prevalence study

**DOI:** 10.4102/sajid.v34i1.129

**Published:** 2019-11-22

**Authors:** Pieter Nel, Lauren A. Roberts, Rena Hoffmann

**Affiliations:** 1 Division of Medical Microbiology, Department of Pathology, Tygerberg Medical Campus, Stellenbosch University, Cape Town, South Africa; 2 Medical Microbiology Laboratory, Tygerberg Hospital, National Health Laboratory Service, Cape Town, South Africa

**Keywords:** Point prevalence, colonisation, carbapenem resistance, carbapenemase, Enterobacteriaceae, bla_NDM-1_

## Abstract

**Background:**

Infections caused by carbapenemase-producing Enterobacteriaceae (CPE) have been increasing worldwide in recent years, but data regarding the prevalence and clinical significance of CPE colonisation in South Africa is not well documented. Local private hospital groups have implemented routine screening programmes for selected high-risk patients as endorsed by the South African Society for Clinical Microbiology. This practice is not routinely performed in the public sector.

**Methods:**

A point prevalence study was performed at Tygerberg Hospital (TBH) by screening patients of all the adult inpatient wards to investigate the current prevalence of CPE colonisation. Common risk factors associated with CPE colonisation were also investigated.

**Results:**

From a total of 439 patient samples collected, only one patient was colonised with a *Klebsiella pneumoniae* organism harbouring bla_NDM-1_. The identified patient had none of the common risk factors associated with CPE colonisation.

**Conclusion:**

Based on these findings, screening for CPE colonisation in adults on admission to TBH is currently not recommended.

## Background

The recent rise in carbapenem resistance has necessitated the screening of high-risk patients to detect colonisation with carbapenemase-producing Enterobacteriaceae (CPE). Enterobacteriaceae is a large group of gram-negative organisms readily found in the environment and in the normal flora of the human gastrointestinal tract. Carbapenem antibiotics are instrumental in treating hospital-acquired infections caused by multi-drug resistant (MDR) Enterobacteriaceae.^1,2,3^ It is apparent that early identification of patients harbouring these MDR organisms in healthcare facilities can assist in the prompt implementation of isolation and cohort-nursing practices with the purpose of minimising the spread and subsequent related morbidity and mortality of these potential pathogens.^4,5,6,7^

The investigators performed a point prevalence study under adult inpatients in Tygerberg Hospital (TBH) with the aim of describing the institutional risk factor profile and to document the risk factors associated with CPE colonisation. This was the first study of its kind to be performed in this institution.

## Materials and methods

### Location

Tygerberg Hospital is a tertiary academic institution located in the Western Cape province of South Africa. It contains approximately 50 adult inpatient wards, with a combined maximum adult bed capacity of approximately 1000. The wards range over different disciplines including internal medicine, surgery, orthopaedics, psychiatry, oncology, intensive care, emergency medicine, obstetrics and gynaecology.

### Study period and population

All the adult inpatient wards were visited once according to a scheduled roster during September – November 2016. The investigators only approached patients that were present in the wards at the time of the visits for inclusion in the study. Patients were selected according to a range of criteria (Table 1), and informed consent was received from all patients in their language of choice (interpreters were used when requested).

**TABLE 1:** Selection criteria for study participation.

Inclusion criteria	Exclusion criteria
Patients older than 18 years	Informed consent refused or withdrawn during the study and patients incapable of granting consent
Informed consent given	Staff and visitors
Inpatients of adult wards including medicine, surgery, orthopaedics, psychiatry, oncology, intensive care, emergency medicine, obstetrics and gynaecology	Day-patient wards, outpatient clinics, theatres and imaging departments were not visited

### Patient samples, risk assessment and anonymisation

Following appropriate counselling, patients received an information leaflet detailing the purpose of the study and were given the choice of submitting a stool sample or having a rectal swab performed. Sample collection was performed by trained investigators according to a standard operating procedure drafted specifically for the purpose of this study. Cotton-wool swabs containing Amies medium gel (Citotest Labware Manufacturing Co., LTD, China) were used to collect the rectal swab samples, and these were stored in a cooler box containing an ice pack for no longer than 3 hours before being processed in the laboratory. Stool samples were collected in disposable stool containers (Citotest Labware Manufacturing Co., LTD, China) and stored in a similar way before laboratory processing. An anonymous risk factor assessment form was completed for each patient who participated in the study. No patient identifiers appeared on the risk factor assessment forms or on the patient samples.

### Microbiological analysis and susceptibility testing

Laboratory health and safety procedures were complied with during all laboratory investigations. Samples were plated onto MacConkey agar (Diagnostic Media Production, Green Point Complex, National Health Laboratory Service, South Africa). An ertapenem disc (Mast Diagnostics, Mast Group Ltd., United Kingdom) was placed at the junction of the main inoculum and the first streak line. Plates were incubated at 37 °C in an ambient incubator for 24 h. The Clinical and Laboratory Standards Institute (CLSI) M100S Performance Standards for Antimicrobial Susceptibility Testing (26th edition of 2016) were used for interpreting the susceptibility results. Following incubation, all suspicious gram-negative colonies growing within the 22 mm zone around the ertapenem discs were either subcultured for single colonies or processed with the Vitek^®^ 2 automated system (bioMérieux, France) for identification and susceptibility testing. All isolates identified as Enterobacteriaceae with elevated minimum inhibitory concentrations (MIC) for any or all the carbapenems were stored for molecular testing.

### Molecular investigations

Isolates that screened positive for resistance to carbapenems on the Vitek^®^ 2 automated system (bioMérieux, France) were processed using a modified protocol based on the method described by Zowawi et al.^8^ The KAPA2G Fast Multiplex PCR Kit (Kapa Biosystems) was used for DNA amplification. Primers for the genes encoding for the NDM-1 (New Delhi metallo-β-lactamase), OXA-48 (Oxacillin-hydrolising carbapenemase 48), VIM (Verona integron-encoded metallo-β-lactamase), IMP (Imipenem-resistant *Pseudomonas*-type carbapenemase) and KPC (*Klebsiella pneumoniae* carbapenemase) enzymes were synthesised by Integrated DNA Technologies. Primers for *rpoB* as described by Hoffmann and Roggenkamp were included as the internal control. A non-template control was used to rule out contamination.^9^ Polymerase chain reaction products were visualised by gel electrophoresis before being sent for sequencing by Inqaba Biotechnical Industries, South Africa. Sequences were analysed with BioEdit Sequence Alignment Editor. Consensus sequences were run through the Basic Local Alignment Search Tool (BLAST) database of the National Centre for Biotechnology Information, United States of America and National Library of Medicine for identification of the specific genes.

### Ethical considerations

This study was approved by the Stellenbosch University’s Health Research Ethics Committee (HREC) (ethics reference: S15/03/064), which complies with the *South African National Health Act* No. 61, 2003 and the United States Code of Federal Regulations Title 45 Part 46. The HREC Committee abides by the norms and principles for research as established by the Declaration of Helsinki, the South African Medical Research Council Guidelines and the Guidelines for Ethical Research: Principles, Structures and Processes 2004 (Department of Health).

## Results

**FIGURE 1:**
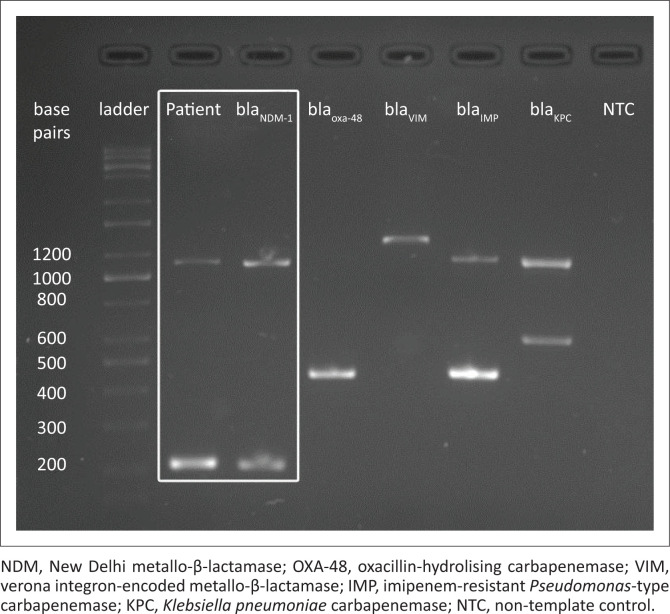
Results of multiplex polymerase chain reaction for carbapenemase-producing Enterobacteriaceae.

A total of 602 adult inpatients were approached during the 3-month study period. Of 448 patients that met the study criteria, nine patients withdrew consent during the sampling process because of personal reasons. Four hundred and thirty-nine patient samples, of which only one was a stool sample, were processed. Twelve samples (12/439, 2.73%) screened positive for carbapenemase resistance. Only one sample (1/439, 0.23%) was identified as Enterobacteriaceae and tested positive for carbapenemase production. The patient in question was a 58-year-old man who was colonised with a *Klebsiella pneumoniae* organism harbouring *bla*_NDM-1_ (Figure 1). He had none of the reported risk factors associated with CPE colonisation.

### Clinical characteristics of patient cohort and risk factor review

The study patients were distributed approximately equally between male (230/439, 52.39%) and female (209/439, 47.61%) gender and had an average age of 44.94 years (range of age: 18–101 years) at the time of sample collection. Twelve risk factors were investigated during this study. The most common risk factor found was the presence of indwelling lines, drains and catheters (158/439, 35.99%). There were 151 (151/439, 34.4%) patients that had concomitant debilitating conditions which included human immunodeficiency virus, tuberculosis, diabetes mellitus, various cancers and auto-immune diseases. One hundred and thirty patients (130/439, 29.61%) underwent recent surgery, and 117 (117/439, 26.65%) were hospitalised in the six months prior to their current hospitalisation. Twenty-three patients (23/439, 5.24%) were recently admitted to an intensive care unit. Twenty-two patients (22/439, 5.01%) were recently exposed to carbapenem antimicrobials (Tables 2 and 3).

**TABLE 2:** Risk factors for carbapenemase-producing Enterobacteriaceae colonisation investigated during the study.

Risk factors	Number of patients with risk factors (*N* = 439)	
	*n*	%
Known previous CPE infection and carriage	0	0
Known exposure to individuals colonised with CPE	0	0
Known debilitating diseases	151	34.4
Recent or current carbapenem exposure	22	5.01
Recent broad spectrum antimicrobial exposure	114	26.65
Recent previous hospital admission	117	26.65
Recent or current ICU admission	23	5.24
Recently or currently ventilated	9	2.05
Recent indwelling lines and catheters and drains	158	35.99
Recent major surgery	130	29.61
Recent exposure to long-term care facility	2	0.46
Recent travels to areas outside South Africa or areas with known high CPE prevalence	8	1.82

CPE, carbapenemase-producing Enterobacteriaceae; ICU, intensive care unit; *N*, denominator; *n*, numerator.

**TABLE 3:** Patients with recent or current antimicrobial exposure.

Antimicrobials	Number of patients exposed (*N* = 439)	
	*n*	%
Colistin (polymyxin E)	2	0.46
Carbapenems	22	5.01
Extended spectrum cephalosporins	23	5.24
Beta-lactam/Beta-lactamase inhibitor combinations	57	12.98
Aminoglycosides	5	1.14
Fluoroquinolones	11	2.51
Vancomycin	10	2.28
Other	53	12.07
Unknown antibiotics	27	6.15
Total number of patients exposed to antibiotics (incl. those exposed to more than one class)	181	41.23

*N*, denominator; *n*, numerator.

## Discussion

Carbapenem resistance among Enterobacteriaceae is conferred through numerous mechanisms of which enzyme production poses the biggest threat.^2,4^ Genes encoding for carbapenemase production mainly occur on genetic elements (plasmids and transposons) found inside the bacteria. This allows for widespread horizontal transfer of resistance mechanisms between different bacterial strains and even species including *Pseudomonas* spp. and *Acinetobacter* spp.^10,11,12,13^

The NDM-1, OXA-48, VIM, GES (Guiana extended spectrum), IMP and KPC enzymes are the most common that have been identified in the public, and private sectors in South Africa.^14^ The first *bla*_NDM-1_ in South Africa and the first *bla*_KPC_ in Africa were both isolated from hospitals in Gauteng in 2011.^15^ Statistics from South Africa’s antimicrobial resistance reference laboratories (AMRRL) revealed that 108 diagnostic isolates sent for CPE investigation between 2012 and 2013 were identified to harbour *bla*_NDM-1_. During the same period, 6% (*N* = 390) of isolates received by AMRRL Cape Town, 48% (*N* = 365) of isolates received by AMRRL Johannesburg and 16% (*N* = 711) of isolates received by a private laboratory in South Africa were positive for CPE.^14^

In TBH, six (42.86%) carbapenem-resistant Enterobacteriaceae isolates from a total of 14 obtained from diagnostic samples were identified to be CPEs during 2013 (institution’s unpublished data).

Despite the steady increase in infections because of CPEs in all African regions, investigation and documentation of CPE colonisation and its clinical significance remain poor.^3^ The lack of data and effective antimicrobials available for treating these extensively drug-resistant bacterial infections are concerning because of the high mortality rate (60% – 80%) associated with infections caused by these organisms.^16,17,18^

The recent implementation of national guidelines by many countries focusing on infection control policies and the timely identification of CPE-related infections and colonisation has sparked a much needed conversation in the South African healthcare system for the need of implementing similar strategies.^4,19,20,21^

Several private hospital groups in South Africa have implemented screening programmes for selected high-risk patients – a practice endorsed by the South African Society for Clinical Microbiology. Interpretation of South African private laboratory data however suggests that the risk factors used for identifying high-risk patients are quite broad and non-specific and generally lead to low screening positivity rates of only 5% – 12%.^20^

The investigators set out to ascertain the infection risk profile of TBH by studying the prevalence of CPE colonisation under adult inpatients. Concurrently, all patients were assessed for specific risk factors generally associated with CPE colonisation as reported in the literature (Box 1).

BOX 1 Known documented risk factors for carbapenemase-producing Enterobacteriaceae colonisation.

**Risk factors**

Previous CPE exposure (contacts of proven cases; exposure whilst in other hospitals or countries where CPE are endemic) and previous broad spectrum antibiotic use

Prior and prolonged hospitalisation and long-term frail care facility admission

Severe illness, intensive care unit admission and surgery

Immunocompromised individuals and those with poor functional status

Mechanical ventilation, dialysis and the presence of invasive devices

Organ and stem cell transplantation

CPE, carbapenemase-producing Enterobacteriaceae.

The prevalence of CPE colonisation in our setting was found to be very low (1/439, 0.23%). A possible reason for this could be that only one screening sample per patient was taken during their hospital stay. It is also noted that the direct molecular screening of patient samples might have identified a higher number of colonised individuals.

Following this point prevalence study, it was apparent that the identified colonised patient had none of the common risk factors associated with CPE colonisation. The investigators also noted that despite the presence of many reported risk factors in the total patient cohort, none of these at-risk patients were colonised with CPE. This finding supports previous reports of poor specificity associated with the current risk factor profile used to screen for CPE colonisation in the South African context.^20^

The conclusion of the investigations is that the routine screening of adult patients for CPE colonisation on admission to TBH is currently unnecessary. We recommend performing follow-up studies in order to monitor the institution’s infection risk profile, as well as to further investigate and define the risk factors associated with CPE colonisation. A similar study investigating the prevalence of CPE colonisation under paediatric inpatients is advised.
